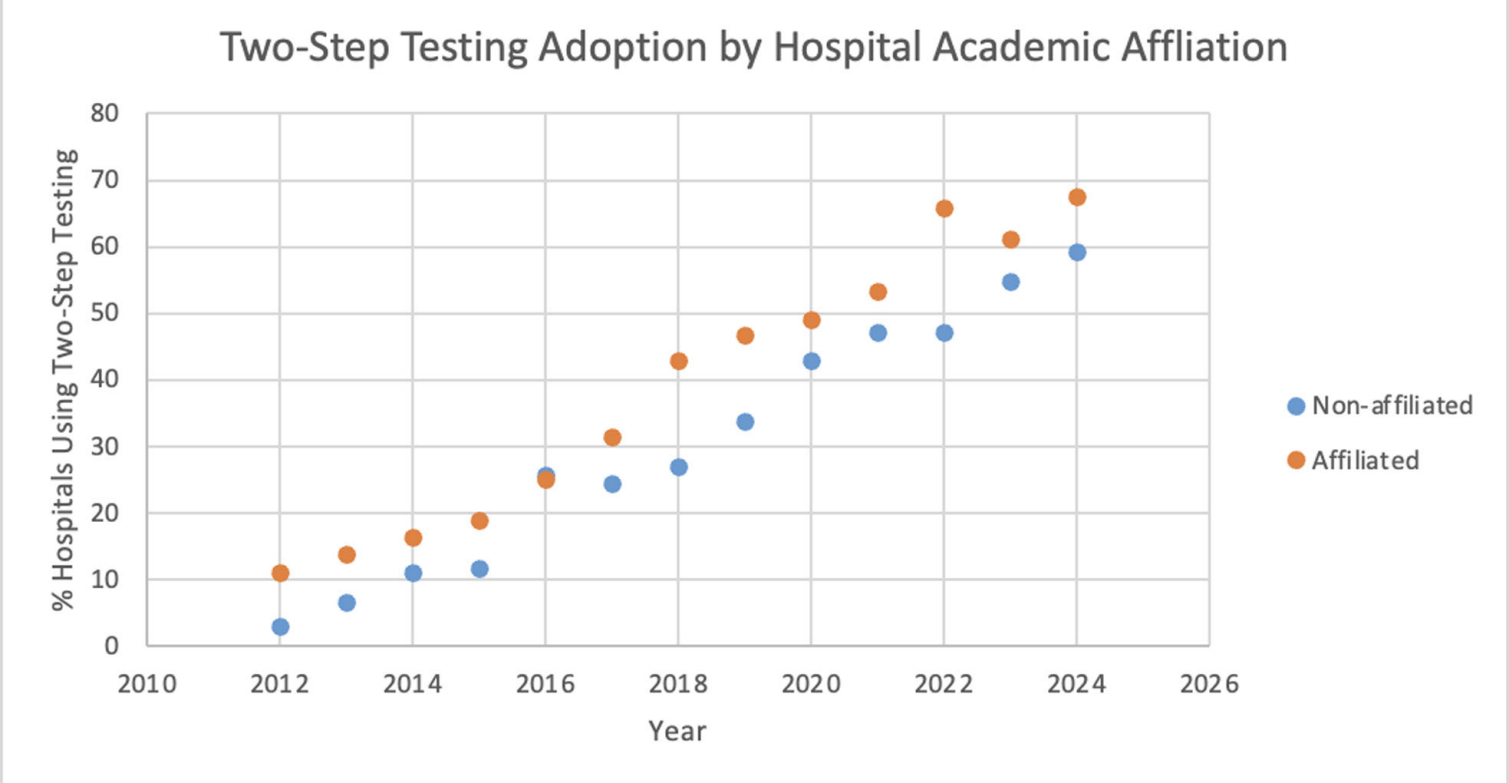# 208 Comparison of MDRO Carriage to Environmental Reservoirs in Ventilator-Capable Skilled Nursing Facilities (vSNFs)

**DOI:** 10.1017/ash.2026.10442

**Published:** 2026-06-23

**Authors:** Sai Pranathi Bingi, Stephanie Stroever, Monika Pogorzelska-Maziarz

**Affiliations:** 1 TTUHSC School of Medicine; 2 Texas Tech University Health Sciences Center

## Abstract

**Background:** Clostridioides difficile infection (CDI) remains a leading healthcare-associated infection (HAI) in the United States. To prevent outbreaks and transmission within healthcare facilities, providers must quickly recognize signs of CDI, correctly diagnose it, and implement measures to contain it. Nucleic acid amplification tests (NAATs) are highly sensitive but may over diagnose CDI, while toxin enzyme immunoassays (EIAs) risk false negatives. Two-step diagnostic algorithms have been recommended to improve diagnostic accuracy; however, national trends in their adoption have not been well described. **Method:** We conducted a secondary analysis of data from the Preventing Infections Through Appropriate Staffing (PITAS) study, which includes annual National Healthcare Safety Network (NHSN) hospital survey data from 347 U.S. acute-care hospitals from 2011-2024. Hospitals reported CDI testing methods annually, which were categorized as one-step, two-step, or other algorithms. Temporal trends in two-step testing adoption were assessed using mixed-effects linear regression with hospital as a random effect and adjustment for bed size and academic affiliation. Stratified analyses were performed by hospital size and academic status. **Result:** A total of 347 acute care hospitals contributed data from 2011-2024. In 2012, the predominant testing method was NAAT alone, which was utilized by 58% of hospitals while only 6.25% of hospitals utilized two-step methodologies. In 2017, the year of publication of the updated Infectious Disease Society of America (IDSA) and Society for Healthcare Epidemiology of America (SHEA) Clinical Practice Guidelines, 28.75% of hospitals used two-step methods. By 2024, 66.18% of surveyed hospitals utilized two-step methodologies for CDI. Academic hospitals consistently demonstrated higher adoption rates than non-academic hospitals (11.11% vs 2.92% in 2012 and 67.35% vs 59.18% in 2024). Hospitals with 501-1000 beds had significantly higher odds of adopting two-step testing compared with the smallest hospitals. **Conclusion:** Use of two-step CDI testing methods increased substantially in U.S. acute-care hospitals from 2011- 2024, particularly following the 2017 IDSA and SHEA guideline update. As hospitals increasingly adopt two-step testing, policymakers and healthcare epidemiologists should consider how testing methods influence reported infection rates.

**Figure:**
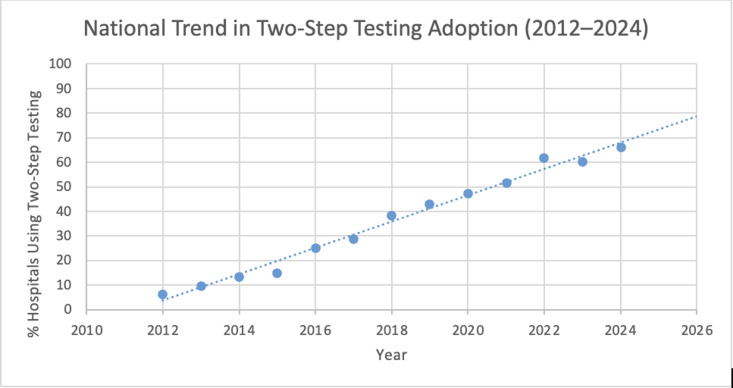

**Figure:**
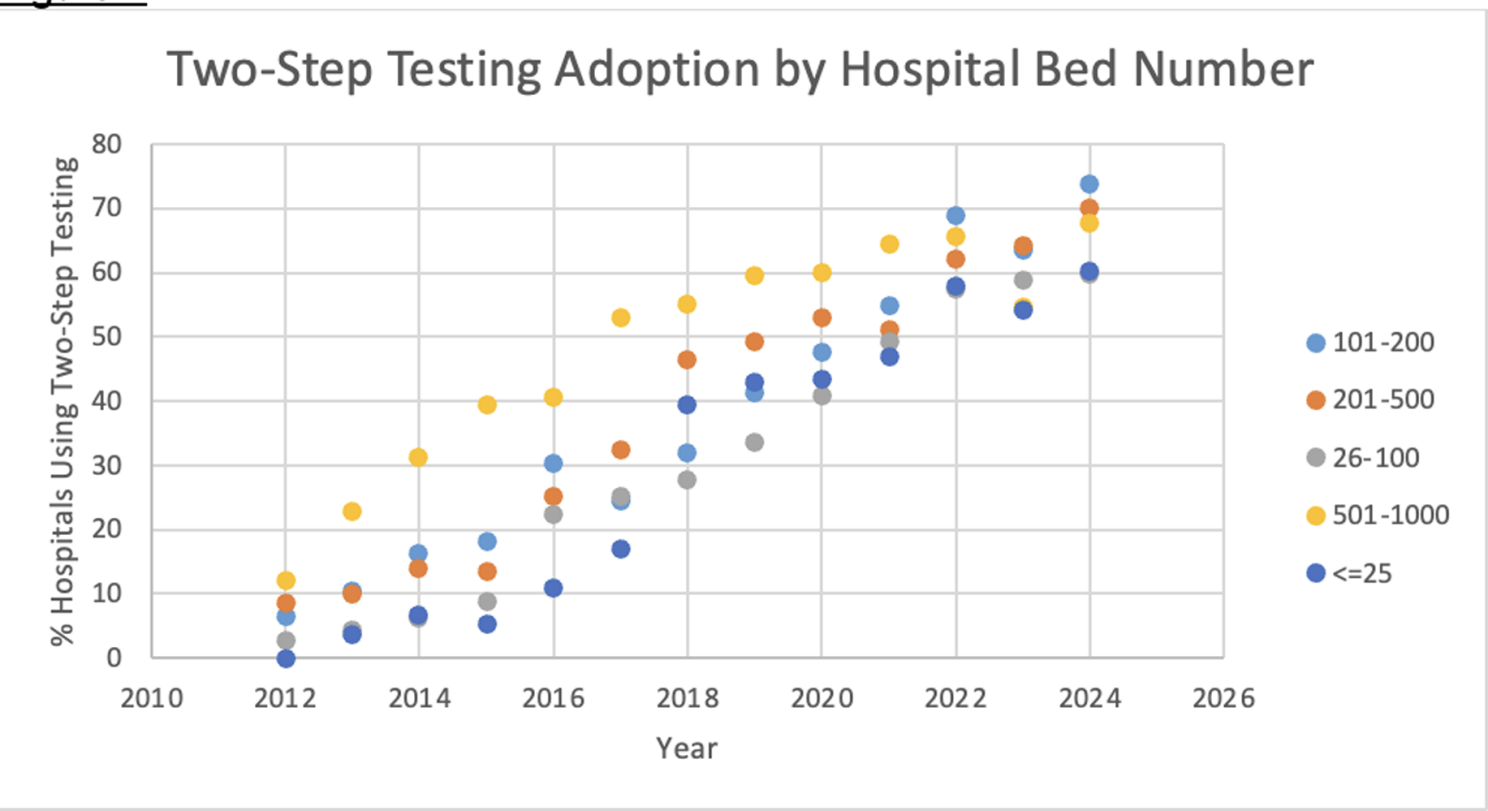

**Figure:**